# Extracellular vesicles from human plasma for biomarkers discovery: Impact of anticoagulants and isolation techniques

**DOI:** 10.1371/journal.pone.0285440

**Published:** 2023-05-10

**Authors:** Valentina Bettio, Eleonora Mazzucco, Annamaria Antona, Silvia Cracas, Marco Varalda, Jacopo Venetucci, Stefania Bruno, Giulia Chiabotto, Chiara Venegoni, Alessandra Vasile, Annalisa Chiocchetti, Marco Quaglia, Giovanni Camussi, Vincenzo Cantaluppi, Massimiliano Panella, Roberta Rolla, Marcello Manfredi, Daniela Capello

**Affiliations:** 1 Department of Translational Medicine, Center of Excellence in Aging Sciences, University of Piemonte Orientale, Novara, Italy; 2 UPO Biobank, University of Piemonte Orientale, Novara, Italy; 3 Department of Medical Sciences, University of Torino, Turin, Italy; 4 Interdisciplinary Research Center of Autoimmune Diseases, Center on Autoimmune and Allergic Diseases, University of Piemonte Orientale, Novara, Italy; 5 Department of Health Science, "Maggiore della Carità" University Hospital, Novara, Italy; 6 Nephrology and Kidney Transplantation Unit, Department of Translational Medicine, University of Piemonte Orientale, "Maggiore della Carità" University Hospital, Novara, Italy; 7 Clinical Chemistry, Azienda Ospedaliera-Universitaria "Maggiore della Carità", Università del Piemonte Orientale, Novara, Italy; Universita Politecnica delle Marche, ITALY

## Abstract

Extracellular vesicles (EVs) isolated from plasma are increasingly recognized as promising circulating biomarkers for disease discovery and progression, as well as for therapeutic drug delivery. The scientific community underlined the necessity of standard operative procedures for the isolation and storage of the EVs to ensure robust results. The understanding of the impact of the pre-analytical variables is still limited and some considerations about plasma anticoagulants and isolation methods are necessary. Therefore, we performed a comparison study between EVs isolated by ultracentrifugation and by affinity substrate separation from plasma EDTA and sodium citrate. The EVs were characterized by Nano Tracking Analysis, Western Blot, cytofluorimetric analysis of surface markers, and lipidomic analysis. While anticoagulants did not significantly alter any of the analyzed parameters, the isolation methods influenced EVs size, purity, surface markers expression and lipidomic profile. Compared to ultracentrifugation, affinity substrate separation yielded bigger particles highly enriched in tetraspanins (CD9, CD63, CD81), fatty acids and glycerolipids, with a predominant LDL- and vLDL-like contamination. Herein, we highlighted that the isolation method should be carefully evaluated prior to study design and the need of standardized operative procedures for EVs isolation and application to biomarkers discovery.

## Introduction

Extracellular vesicles (EVs) are membrane-delimited vesicles that can be released from any cellular lineage [1, 2]. Based on their size and biogenesis they are traditionally distinguished in microvesicles (MVs), produced by the outward budding of the plasma membrane, exosomes, generated through the fusion of multi vesicular bodies with the plasma membrane, and apoptotic bodies, larger vesicles generated during the membrane blebbing and consequent vesiculation of apoptotic cells [2–5]. However, since information about markers specifically associated to each EVs population are still fragmentary, the International Society of Extracellular Vesicles strongly suggest to use the generic term of EVs [6, 7].

EVs are recognized as mediators of cell-to-cell communication due to their ability to transfer biomolecules among cells and to influence the extracellular microenvironment [8–10]. EVs not only serve several physiological functions [10–12], but many pieces of evidence suggest also their role in the pathophysiology of inflammatory and degenerative diseases [13–16].

EVs cargo is vast and consists of nucleic acids, cytokines, enzymes, growth factors, functional organelles (e.g., proteasome, mitochondria) and transcription factors [2, 17, 18]. Such a rich repertoire of cargo has boosted the interest in EVs as a potential source of clinical biomarkers [19–21].

The EVs lipid content, instead, has been poorly investigated. The lipid composition of EVs membranes affects their stability in the extracellular environment and the interaction with target cell, and represents a source of molecules that could trigger paracrine signals [2, 22]. Interestingly, the EVs lipid pattern was found to be modified in pathological conditions and it is emerging as a new important source of biomarkers and indicators of health status [23–27].

Plasma and serum are major sources of EVs for clinical applications, since liquid biopsies represent a low-invasive procedure [28]. Moreover, blood hosts EVs secreted not only by leukocytes, platelets, erythrocytes and endothelial cells [10, 29, 30], but also by other cell types, in both physiological and pathological conditions [36–38]. Thus, serum and plasma are attractive sources of EVs that represent a good surrogate for the identification of informative biomarkers for disease diagnosis, prognosis and longitudinal monitoring [31, 32].

Fresh unmanipulated blood would be the more reliable source of EVs for diagnostic purposes, and recently a fast, cheap and easy method to detect EVs, in few microliters of fresh peripheral blood by flow cytometry, has been established [33] and validated [34]. Nevertheless, the literature agrees that plasma is a more adequate source of EVs, since additional platelet-derived EVs are released during clot formation while preparing serum [35]. Despite the increasing interest, the isolation of EVs from human plasma is hampered by the limited availability of starting material and by the lack of suitable EVs separation procedures capable of efficiently reducing non-vesicular macromolecules contamination [36, 37]. Moreover, multiple pre-analytical variables can affect the quality and the yield of purified EVs (e.g. anticoagulant, manipulation during transportation, storage, isolation methods) [35, 37–42]. In particular, studies investigating anticoagulants on EVs separation reported conflicting results [38, 39, 41, 43]. Today the choice of the anticoagulant is still based on the downstream applications, and the most commonly used are sodium-citrate, especially for study platelet-derived EVs, and EDTA for genomic and proteomic analysis [44–46].

Despite the increasing interest in EVs as a source of clinically relevant biomarkers, no official guidelines for EVs preparation for diagnostic purposes are still available. At present, ultracentrifugation (UC), eventually in presence of a density gradient, and size exclusion chromatography (SEC) represent the gold standard for EVs isolation for research purposes [47]. Although these two methods ensure an unbiased isolation of EVs, UC suffers from important limitations affecting the purity, the aggregation and the integrity of EVs [48, 49], whereas EVs obtained by SEC usually need a further concentration step and are not depleted from contaminants [50, 51]. Moreover, both methods are time consuming, not scalable and can hardly be integrated into routine diagnostics. On the other hand, faster and more scalable approaches, such as immunoaffinity-based capture strategies, are highly expensive and give a pre-cleared preparation that is not representative of the whole EVs population [51]. Interestingly, a new approach for EVs isolation based on affinity substrate separation (AS) has been developed (ExoEasy, Qiagen) [52]. AS is a fast and scalable method of isolation, showed higher yields of EVs with respect to SEC and can be directly used for RNA and miRNA extraction, offering a linear workflow and a simple protocol for the downstream analysis of RNA and miRNA, the most promising EVs-related markers up today [53–57].

Frozen biobanked plasma, serum and other body fluids are a valuable source of EVs for retrospective studies and biomarkers identification for clinical applications [58, 59]. Here, we evaluated the impact of the main anticoagulants employed in our Institution (sodium citrate and potassium-EDTA) on plasma EVs profile focusing our analysis on the number, diameter, purity, and protein and lipid composition. Moreover, we tested and compared two methods for EVs isolation from small plasma volumes, since biobanked plasma samples are typically stored in 100–500 μl aliquots. At first we used UC, a method that would not introduce bias into purified EVs populations and would provide sufficient yield for downstream analysis. Then we investigated AS as a potentially valuable method in place of UC for downstream immunophenotypic and lipidomic characterization of EVs.

Our data showed no significant differences between EVs isolated from citrate and from EDTA neither in terms of quality of the preparation nor in terms of lipidomic profile. Remarkably, significant differences in surface markers and lipid composition have been observed between EVs isolated by UC and EVs isolated by AS, pointing to the importance of careful study design and data interpretation in studies using blood-derived EVs with special focus on potentially co-purified contaminants, such as lipoproteins.

## Materials and methods

### Participants, ethical approval and blood sample processing

This research has been conducted using UPO Biobank biological specimens derived from participants of the BioMAge project (https://biobank.uniupo.it/) approved by the Institutional Ethics Committee (EC number 290/20). Blood samples were derived from 30 healthy subjects fitting the following inclusion criteria: male sex, age range between 25 and 50, free of any major disease and taking no medications.

All blood samples were processed by UPO Biobank specialized operators, as specified in S1 File. For the study, each sodium citrate and EDTA plasma pool (Pool A, Pool B, Pool C, Pool D, and Pool E) were prepared pooling PPP from six different individuals.

### Isolation of EVs

EVs were isolated from both sodium citrate and EDTA pooled plasma by UC (Optima™ LE-80K Ultracentrifuge, Beckman Coulter) and affinity substrate isolation (AS) using the ExoEasy kit (Qiagen). Before EVs isolation, the pooled plasma (Pool A, Pool B, Pool C, Pool D, and Pool E) were clarified by centrifugation at 3000 g for 15 min at 4°C. EVs were then isolated by UC as follow: 1.0 mL of sodium-citrate or EDTA pooled plasma were diluted 1:12 in filtered, cold PBS and centrifuged at 146,000 g for 2 hours at 4°C. After discarding the supernatant, the pellets were suspended into 600 μL of filtered, cold PBS, aliquoted into 1.5 mL tubes and stored at -80°C. We have submitted all relevant data of our experiments to the EV-TRACK knowledgebase (EV-TRACK ID: EV230053) [60].

The isolation of EVs with the ExoEasy kit (Qiagen) has been performed following the manufacturer’s instructions starting from 1.0 mL of sodium-citrate or EDTA pooled plasma and by eluting EVs with 600 μL of the elution buffer supplied in the kit.

### NTA analysis

EVs concentration and size distribution were determined by using the NanoSight NS300 (Salisbury, UK) equipped with a 488 nm blue laser and a sCMOS camera. A camera level 10–13 was used for data acquisition, as reported in SOP Standard Measurement. Data were analyzed with NanoSight NTA 3.3 software with detection threshold 4. The analysis allowed the determination of EVs concentration and size based on the EVs Brownian movements and by applying the Stokes-Einstein equation. For each sample three videos of 30 seconds duration were recorded. Before analysis, samples were properly diluted with filtered PBS. Mean values for concentration and size distribution were calculated. Statistical analysis was performed by using Graphpad Prism version 7.0 (GraphPad Software, San Diego, California USA).

### Transmission electron microscopy (TEM)

Transmission electron microscopy was performed on EVs isolated by UC and AS on 200 mesh nickel formvar carbon-coated grids (Electron Microscopy Science, Hatfield, PA, USA). After an adhesion step (20 minutes), the grids were incubated with 2.5% glutaraldehyde containing 2% sucrose and extensively washed in distilled water. Finally, the EVs were negatively stained with NanoVan (Nanoprobes, Yaphank, NK, USA) and acquired with a Jeol JEM 1010 electron microscope (Jeol, Tokyo, Japan).

### SDS-PAGE Western blot analysis of EVs protein markers

The isolated EVs were lysed using RIPA lysis buffer (25 mmol/L Hepes pH 8, 135 mmol/L NaCl, 5 mmol/L EDTA, 1 mmol/L EGTA, 1 mmol/L ZnCl_2_, 50 mmol/L NaF, 1% Nonidet P40, 10% glycerol) with protease inhibitors (AEBSF, aprotinin, bestatin, E-64, EDTA, leupeptin, Sigma-Aldrich) and orthovanadate, assisted by mechanical lysis on a wheel, for 20 minutes at 4°C.

Proteins in EVs lysates were measured using the Pierce BCA protein assay kit (Thermo Fisher Scientific) and 18 μg of proteins for each sample were loaded into polyacrylamide gels. Proteins were denatured at 95°C for 5 minutes in the presence of 2% Sodium Dodecyl Sulfate (SDS), 150 mmol/L dithiothreitol (DTT), and 0.1% bromophenol blue. Proteins were separated by SDS-PAGE using 8% and 15% polyacrylamide gels and transferred to a polyvinylidene difluoride membrane (PVDF, Amersham). PVDF membranes were saturated at room temperature for 1 hour with blocking buffer consisting in Tris Buffered Saline 1X (TBS, Trizma base 50 mmol/L, NaCl 120 mmol/L), 0.1% Tween-20, 3% Bovine Serum Albumin (BSA, Sigma), and incubated 18 hours at 4°C with the primary antibodies dissolved in blocking buffer with 0.01% sodium azide. The primary antibodies were removed and the membranes were washed three times with washing buffer (TBS 1X, 0.1% Tween-20) for 15 minutes and then incubated at room temperature for 1 hour with horseradish peroxidase conjugated secondary antibodies (Perkin Elmer Life Science) diluted 1:3000 in washing buffer. Then, membranes were washed three times with washing buffer for 15 minutes and chemoluminescent signals acquired with the Chemidoc Touch (BioRad) using the ECL Western Lightning Chemiluminescence Reagent Plus (Perkin Elmer Life Science).

### MACSPlex human exosome kit

Before the analysis, AS-EVs were concentrated and desalted using an Amicon Ultra (AU) filter with a cut-off 10K MWCO.

EVs isolated by both UC and AS were subjected to a bead-based flow-cytometry analysis using the MACSPlex Exosome Kit human (Miltenyi Biotec) following the manufacturer’s instruction, as reported in S1 File.

Samples were detected with the Attune NxT flow-cytometer (Thermo Fisher Scientific). Data were analyzed using the FlowJo software package (Tree Star Inc., Ashland, OR, USA), following the manufacturer’s instructions, and Graphpad Prism 7.0 version (see S1 File).

Heath maps were generated by MetaboAnalyst 5.0 software, applying original data, autoscale sample, Euclidean distance measure, Ward clustering method and default color contrast. Statistical analysis was performed by Graphpad Prism version 7.0. Graph error bars showed normalized MFI standard deviations.

### Lipids extraction from EVs and from plasma

Lipids extraction was carried out using a biphasic method, as reported from Barberis et al. [61] and in S1 File.

### Untargeted lipidomic analysis

The reconstituted samples were analyzed by an UHPLC Vanquish system (Thermo Fisher Scientific) coupled with an Orbitrap Q-Exactive Plus (Thermo Fisher Scientific). The separation of lipids was achieved by a reverse phase column (Hypersil Gold™ 150 × 2.1 mm, particle size 1.9 μm) maintained at 45°C at a flow rate of 0.260 mL/min.

Mass spectrometry analysis was performed in both positive and negative ion mode. Lockmass and regular inter-run calibrations were used for accurate mass-based analysis. An exclusion list for background ions was generated analyzing the same procedural blank sample, both for the positive and negative ESI mode. To avoid possible bias during the statistical analysis, sequences of samples were independently randomized for the isolation, extraction, and LC-MS analysis.

Additional information is available in S1 File.

### Lipidomic data processing

The acquired raw data from the untargeted analysis were processed using the MS-DIAL software 4.24 version (Yokohama City, Kanagawa, Japan), as reported from Tsugawa et al. [62]. This included the detection of peaks, MS2 data deconvolution, compound identification, and the alignment of peaks through all the samples. For identification, a cut off value of 85% was selected.

For quantification, the peak area of the different detected molecular species for each lipid were combined (e.g., [M+H]^+^ and [M+NH_4_]^+^ and [M+Na]^+^ and [M+H−H_2_O]^+^ for positive ion mode, and [M−H]^-^ and [M+CH_3_OO]^-^ in negative ion mode). Finally, an in-house library of standards was used for lipids identification. Lipids names and classification were assigned by using the MS-DIAL annotation code [63]. Five replicates for each sample were analyzed. Average lipid species areas were normalized for the total area and for the number of particles determined by NTA. Dataset elaboration and statistical analysis were performed by using MetaboAnalyst version 5.0 and Graphpad Prism version 7.0. Additional information is available in S1 File.

## Results

### EVs preparations

In this study, we evaluated the impact of anticoagulants on EVs preparations by comparing EVs purified from blood collected in sodium citrate (cit) and EDTA. For EVs purification, we employed and compared two different methods: ultracentrifugation (UC), the gold standard method recommended for obtaining high-yield, unbiased EVs preparations [7, 64], and a standardized, time-saving, affinity-based separation procedure (AS) [52]. A total of 5 pooled plasma with citrate and 5 matched pooled plasma with EDTA were prepared from plasma samples obtained from 30 healthy males and stored at -80°C in UPO Biobank for at least 4 weeks (Table 1).

**Table 1:** Characteristics of plasma pools.

				EVs size media (SD)				(EVs number/mL)/(μg protein/mL) ratio			
				UC		AS		UC		AS	
Pool	n° of subjects	Sex	Age mean (SD)	Citrate	EDTA	Citrate	EDTA	Citrate	EDTA	Citrate	EDTA
**Pool A**	6	Male	40 (6)	154.1 (3.2)	170.3 (10.4)	201.6 (2.8)	222.6 (1.0)	1.8*10^7^	2.0*10^7^	5.5*10^7^	4.7*10^7^
**Pool B**	6	Male	38 (6)	133.6 (1.2)	107.3 (1.5)	235.6 (5.4)	251.7 (1.5)	4.8*10^7^	2.5*10^7^	5.9*10^7^	5.2*10^7^
**Pool C**	6	Male	33 (8)	210.5 (5.6)	158.4 (0.9)	243.0 (5.7)	249.0 (3.5)	6.8*10^7^	1.8*10^7^	5.2*10^7^	4.5*10^7^
**Pool D**	6	Male	34 (8)	156.4 (2.0)	173.5 (6.6)	235.1 (0.9)	242.6 (2.9)	1.2*10^7^	1.7*10^7^	6.2*10^7^	4.8*10^7^
**Pool E**	6	Male	34 (3)	157.7 (4.5)	149.9 (2.0)	232.5 (1.7)	249.7 (2.4)	3.7*10^7^	1.1*10^7^	4*10^7^	2.6*10^7^

Each pool was prepared by mixing plasma derived from 6 individuals. From each pool, EVs were isolated by UC (UC-EVs_cit and UC-EVs_EDTA) and by AS (AS-EVs_cit and AS-EVs_EDTA).

### NTA analysis

**Fig 1:**
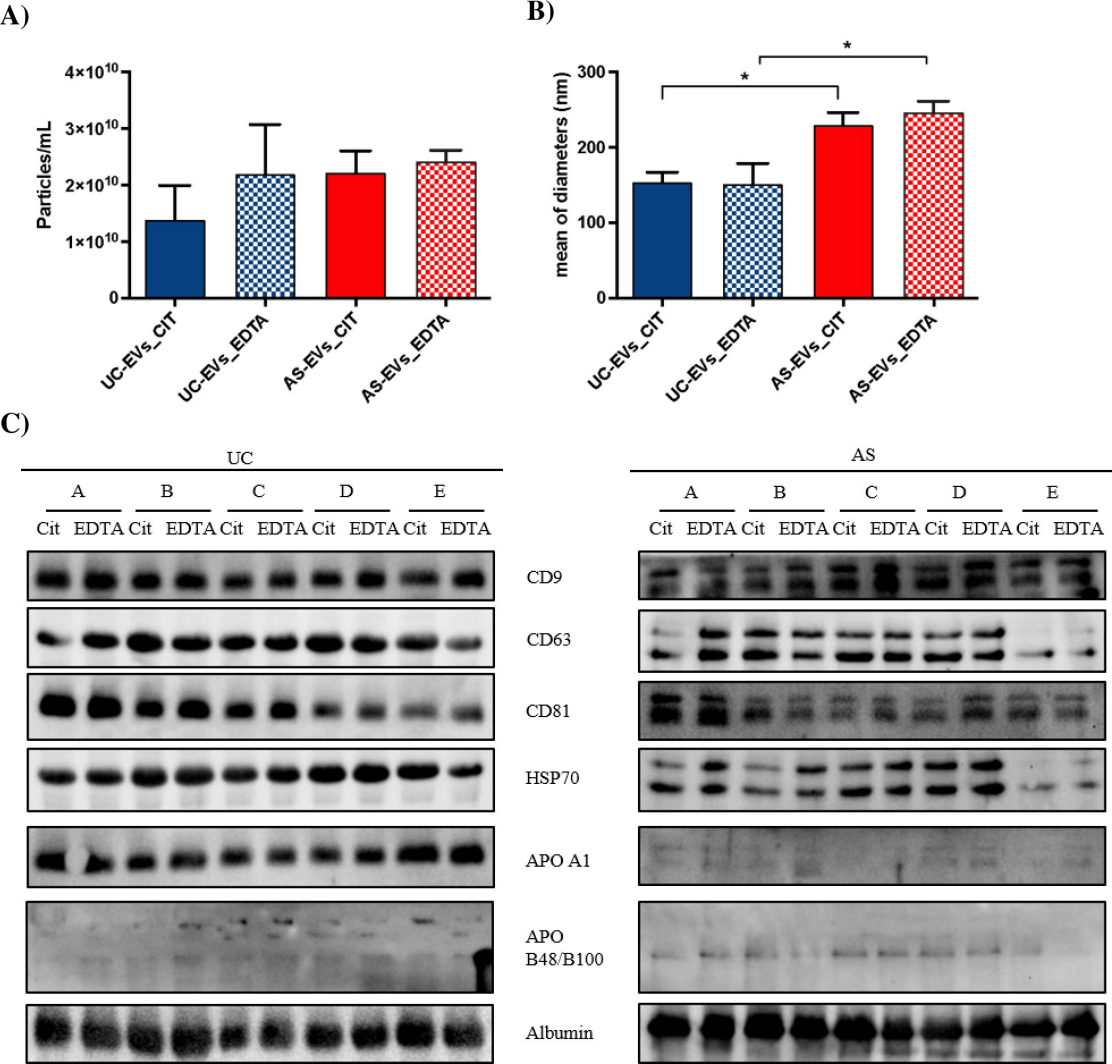
Characterization of the EVs isolated by UC and AS. (A) EVs quantification by NTA (particles/mL; mean ± SD; n = 3 independent reading for each condition). (B) Diameter of particles measured by NTA (mean ± SD; n = 3 independent reading for each condition). *, Student’s T-test, p< 0.05. (C) Western blot analysis of the EVs markers (HSP70 and CD9, CD63 and CD81 tetraspanins) and plasma contaminants (APOA1, APOB48/B100 and albumin) in UC- and AS-EVs.

EVs count by NTA analysis revealed a similar EVs concentration in AS-EVs_cit and AS-EVs_EDTA (2.22 x 10^10^ and 2.42 x 10^10^ particles/mL, respectively), whereas a tendency, although not statistically significant, to a lower EVs yield was observed in UC-EVs_cit (1.38 x 10^10^ particles/mL) when compared with UC-EVs_EDTA (2.19 x 10^10^ particles/mL) (Fig 1A). The overall vesicular structure of the isolated UC- and AS-EVs was preserved, as demonstrated by TEM, despite a reduced general quality and integrity of the membranes (S1 and S2 Figs, inserts). Analyzing the size distribution, significant differences were observed when considering the isolation method but not the anticoagulant type. Indeed, the mean EVs diameter was significantly larger (p<0.05) in AS-EVs_cit (230±42 nm) and AS-EVs_EDTA (246±56 nm) when compared with UC-EVs_cit (154±12 nm) and UC-EVs_EDTA (152±27 nm) respectively (Fig 1B; S1 and S2 Figs), suggesting that UC and AS isolate different EVs-populations.

### EVs protein markers characterization

We thus evaluated the presence of typical protein EVs markers by western blot analysis in both UC- and AS-EVs, in agreement with the MISEV2018 guidelines [7]. In particular, we analyzed the presence of EVs markers belonging to categories 1 and 2 (i.e., CD9, CD63, CD81, HSP70), and non-EVs-markers listed in categories 3 and 4 (i.e., albumin, APO A1, APO B48-100, Histones) [7] (Fig 1C and 1D). Western blot analysis of tetraspanins showed a similar enrichment of CD9, CD63, and CD81 in UC-EVs_cit and UC-EVs_EDTA, as well as in AS-EVs_cit and AS-EVs_EDTA. The presence of tetraspanin bands with different sizes in AS-EVs, is probably due to alterations of post-translational modifications, such as glycosylation, as previously reported [65].

The absence of histones confirmed that the pre-centrifugation step, applied during plasma clearing, was sufficient to deplete apoptotic bodies and free nuclei from our samples (S3A Fig). The presence of albumin was an indication of plasma protein contamination in both AS and UC-purified EVs. Apolipoprotein A1 (APO A1), a marker of high density lipoproteins (HDL) contamination, was highly enriched in UC-EVs compared with AS-EVs, whereas the chylomicrons and low density lipoproteins (LDL) marker apolipoprotein B-48/B-100 (APO B48/B100), was weakly enriched in AS-EVs (Fig 1C). The presence of a strong contamination by non-EVs plasma proteins in both preparations was confirmed by a particles/protein ratio lower than 3x10^10^ for UC-EVs (Table 1) [66].

### Multiplex phenotyping of UC- EVs and AS-EVs

**Fig 2:**
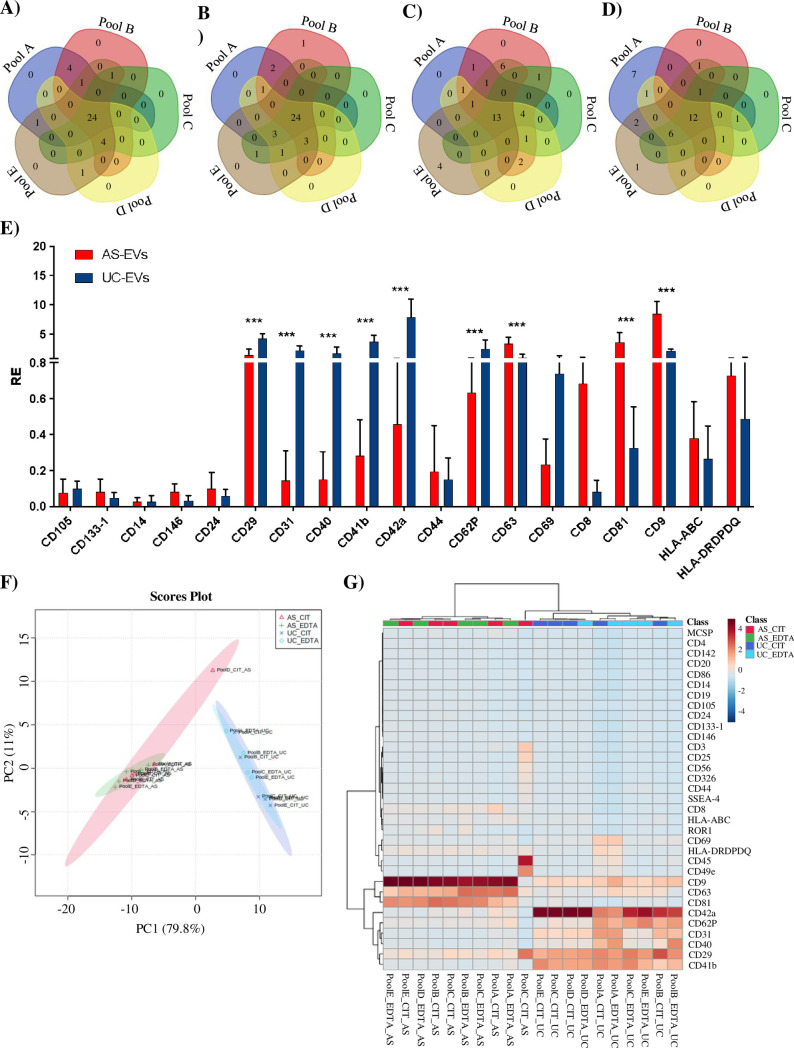
Characterization of the surface epitopes of UC- and AS-EVs by using the MACSPlex kit. Venn diagrams showing the overlap of the markers identified with the MACSPlex kit in UC-EVs_cit (A), UC-EVs_EDTA (B), AS-EVs_cit (C) and AS-EVs_EDTA (D). (E)Histograms showing the normalized MFI of the 19 markers shared by UC- and AS-EVs (mean ± SD). ***, Student’s T-test, p< 0.001. (F)PCA score plot for the markers detected by the MACPlex kit illustrating the relationship between the different EVs preparations. UC-EVs_cit = blue, UC-EVs_EDTA = light-blue, AS-EVs_cit = red, AS-EVs_EDTA = green. Circles represent the 95% confidence interval. (G) Heatmap of the unsupervised hierarchical clustering of surface epitopes investigated with the MACSPlex kit in UC- and AS-EVs. Autoscaling samples and Euclidean distance measure were applied.

To better characterize the surface marker profile of UC- and AS-EVs, we performed a multiplex bead-based cytometry (MACSPlex exosome plasma kit, Miltenyi Biotec), that allows the simultaneous detection and semi-quantitative analysis of 37 different EVs surface epitopes. The mean fluorescence intensity (MFI) of every single marker was analyzed as reported in material and methods. Overall, 29/37 (78.4%) markers were expressed in at least 50% of investigated EVs pools, 3 (CD1c, CD2, and CD11c) were found in less than 20% of samples, whereas the dendritic cell lectin CD209, was undetectable (S1 Table). Venn diagrams, showing the markers distribution among pools, confirmed the homogeneity of UC-EVs pools composition, with 24 (64.9%) and 21 (56.7%) out of 37 markers shared by all UC-EV_cit and UC-EVs_EDTA pools, respectively (Fig 2A and 2B). A lower homogeneity in marker expression was observed among AS-EVs pools, with 13 (35.1%) and 12 (32.4%) out of 37 markers shared by all AS-EV_cit and AS-EVs_EDTA pools, respectively (Fig 2C and 2D). Consistently, the number of markers expressed in at least 60% (the threshold to consider the epitope to be significantly expressed) EVs pools of the same type, i.e. derived from the same anticoagulant and separation method, was higher in UC-EVs (28/37; 78.4%) than in AS-EVs (21/37; 56.8%) (S1 Table). When we compared the MFI of markers shared by UC-EVs_cit and UC-EVs_EDTA (N = 28) as well as of that shared by AS-EVs_cit and AS-EVs_EDTA (N = 19), no significant differences in MFI expression were observed between EDTA- and citrate-EVs pool (S3B and S3C Fig). On the contrary, analysis of markers shared by AS- and UC-EVs revealed significant differences in MFI (Fig 2E). A significant enrichment of platelet- (i.e. CD41b, CD42a, CD62P), T and B lymphocytes- (i.e. CD29, CD40), and endothelium-associated markers (i.e. CD31) was observed in UC-EVs. On the other hand, and as expected [75], AS-EVs were significantly enriched in EV-associated tetraspanins (i.e. CD9, CD63 and CD81), and in CD8, when compared with UC-EVs.

These data show that EVs populations isolated from plasma are not affected by the anticoagulant, but, instead are strongly impacted by the purification method. Principal component analysis (PCA), confirmed the clear distinction between UC-EVs and AS-EVs, independently from the anticoagulant used (Fig 2F), whereas an unsupervised hierarchical clustering of UC- and AS-EVs was clearly outlined and highlighted an expression profile that undoubtedly distinguishes the two EVs preparations (Fig 2G). UC-EVs were characterized by a preferential expression of B-cells, T-cells and leukocytes (i.e. CD20, CD40, CD44, CD69, CD86, and ROR-1), platelets and megakaryocytes (i.e. CD41b, CD42a, and CD62p), epithelial, endothelial, mesenchymal (i.e. CD29, CD31, CD105, CD326), and stemness (i.e. CD24, and SSEA-4) markers. Tetraspanins (i.e. CD9, CD63, and CD81), T-cells and leukocytes (CD8, and HLA-DRDPDQ) and CD146 stemness markers were preferentially detected in AS-EVs instead. These observations suggest that AS- and UC-EVs are represented by different EVs populations as regards both the type of vesicles (microvesicles or exosomes) and the cell of origin, with AS-EVs enriched in tetraspanin, which are usually referred to as exosomal markers.

### The lipidomic profile of AS- and UC-EVs

**Fig 3:**
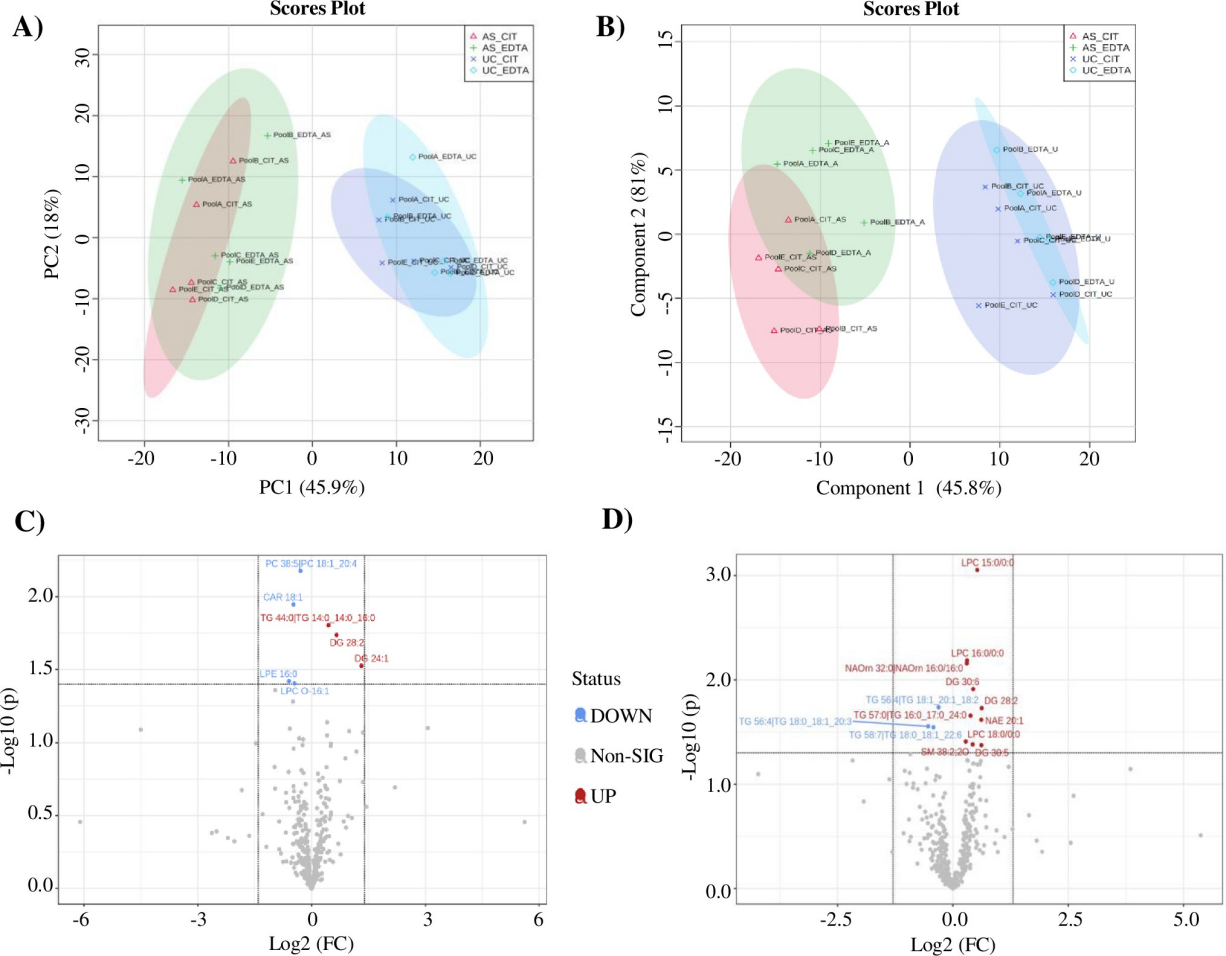
Statistical analysis of the lipidomic data of the isolated EVs. PCA (A) and PLS-DA (B) score plots for the lipids detected by untargeted lipidomic analysis displaying the separation between and UC- (blue and light-blue) and AS-EVs (red and green) and no separation between EVs_cit and EVs_EDTA, when purified with the same isolation method. Circles represent the 95% confidence interval. Volcano plot showing the lipid species that are statistically different (Student’s T-test, p< 0.05) between UC-EVs_cit and UC-EVs-EDTA (C) and between AS-EVs_cit and AS-EVs_EDTA (D). The X-axis represents the Log2-transformed FC and the Y-axis represents the log10-transformed p-value. In red the species that are significantly more expressed in citrate, in blue the species significantly more expressed in EDTA.

**Fig 4:**
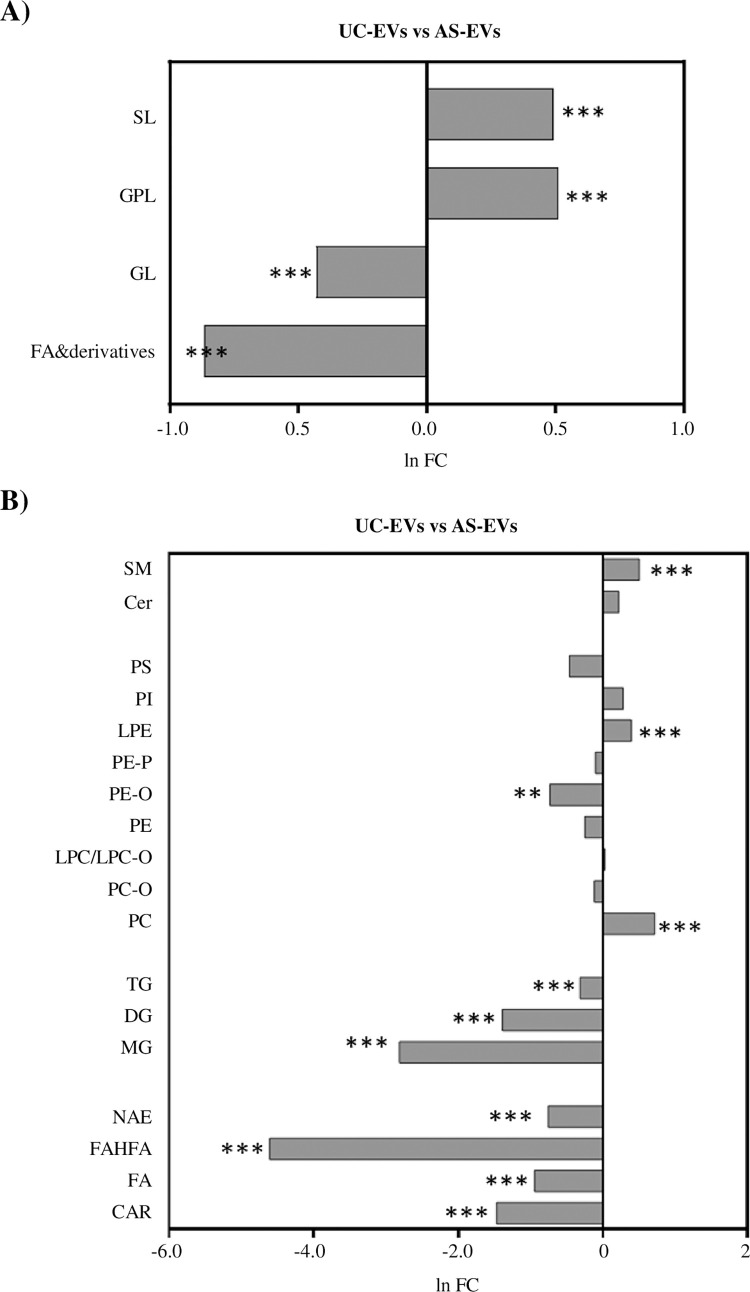
Analysis of the main lipid categories and classes regardless of the anticoagulant. (A) Fold change (FC) of the main lipid categories in UC- versus AS-EVs. All the classes were statistically different, SL and GPL higher in UC-EVs while GL and FA and derivatives higher in AS-EVs. ***, Student’s T-test, p< 0.001. (B) FC and statistical analysis for each lipid class (UC- versus AS-EVs). **, Student’s T-test, p< 0.01; ***, Student’s T-test, p< 0.001.S3.

**Fig 5:**
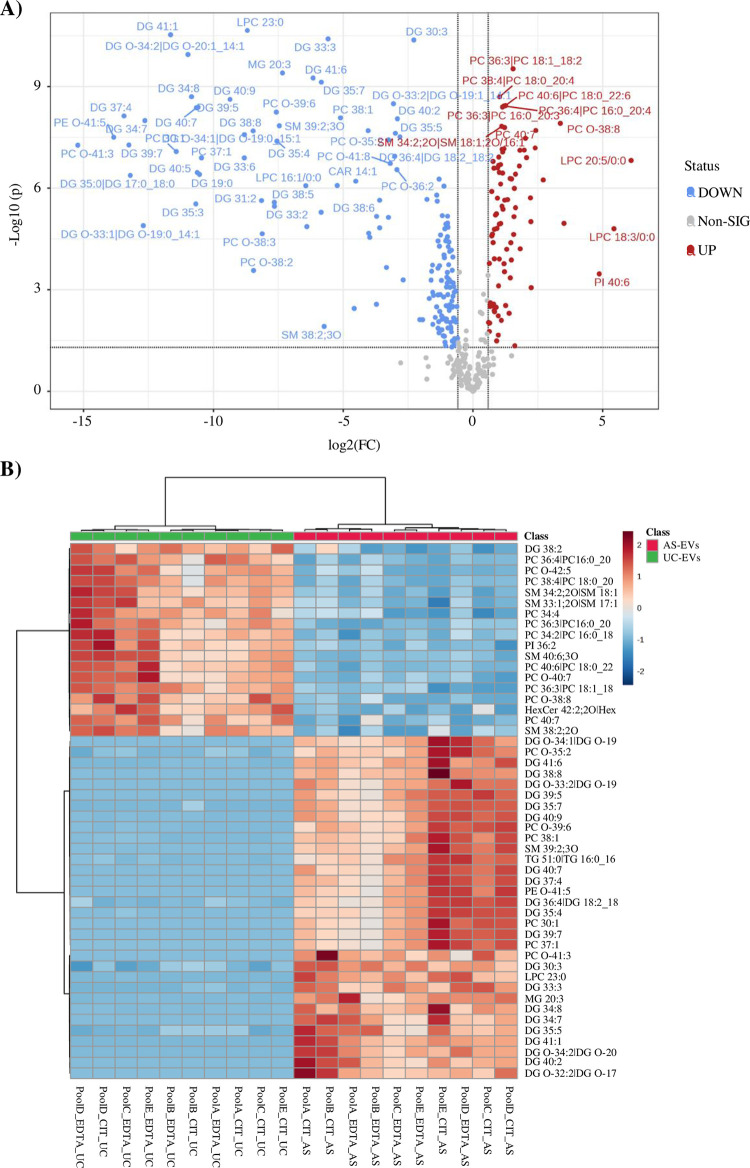
Characterization of the main lipid categories and classes regardless of the anticoagulant. (A) Volcano plot showing the lipid species that are statistically different (fold change> 1.5; Student’s T-test, p< 0.05) between UC- and AS-EVs. The X-axis represents the Log2-transformed FC and the Y-axis represents the log10-transformed p-value. In red the species that are significantly more expressed in UC-EVs, in blue the species significantly more expressed in AS-EVs. (B) Heatmap of the unsupervised hierarchical clustering of the top 50 deregulated lipid species in order of p-value (UC-EVs versus AS-EVs) detected by untargeted lipidomic analysis in UC- and AS-EVs.

The characterization of lipid composition can help to better define EVs populations and identify novel biomarkers. EVs lipidomic analysis was performed with an untargeted approach on UC- and AS-EVs purified from both citrate and EDTA pools. From the data processing of mass spectrometry results, 24 lipid classes (defined by head group) and 367 species (defined by the head group, fatty acid tail length, and saturation) were identified across all samples (S2 Table). EVs pools derived from the same anticoagulant and separation protocol shared more than 88% lipid species, confirming the consistency of pools composition and isolation method (S4 Fig). Investigation of lipid species distribution based on PCA and Partial Least Squares Discriminant Analysis (PLS-DA) showed a clear separation between AS- and UC-EVs, whereas no differences were observed between EVs_cit and EVs_EDTA, when purified with the same isolation method (Fig 3A and 3B). Analysis of normalized areas confirmed the highly similar lipidomic profile of EVs pools derived from different anticoagulants but purified by the same method. No statistically significant differences were observed when we compared lipid categories and classes (S4 Fig) whereas, when we considered the lipid species, only 7 (1.91%) and 13 (3.54%) out of 367 were significantly different between citrate and EDTA EVs pools when purified by UC or AS, respectively (Fig 3C, 3D). Given the almost total identity of EVs_cit and EVs_EDTA, we further characterized the lipid profile of UC- and AS-EVs by analyzing the main lipid categories and classes, regardless of the anticoagulant used (Fig 4A and 4B). A significant enrichment of fatty acids (FA) and derivatives (2.4-fold increase, p<0.001) and glicerolipids (GL) (1.5-fold increase, p<0.001) was observed in AS-EVs, whereas glicerophospholipids (GPL) and sphingolipids (SL) were enriched in UC-EVs (1.67- and 1.64-fold increase, p<0.001) (Fig 4A). When we examined the single lipid classes, UC-EVs were characterized by a significant enrichment of sphingomyelins (SM), lysophosphatidylethanolamine (LPE) and phosphatidylcholine (PC) (Fig 4B; S5 Fig). On the contrary, AS-EVs showed significant enrichment in ether-linked phosphatidylethanolammine (PE-O), in all major classes of FA and derivatives, and in all major classes of GL (Fig 4B; S5 Fig). Next, we got an insight into the alterations of the individual lipid species, analyzing the distribution and enrichment of the species according to the EVs purification method. A total of 244 lipid species showed more than 1.5-fold change (FC) and p-value <0.05 (Fig 5A). The unsupervised hierarchical clustering in Fig 5B displays the top 50 deregulated lipid species in order of p-value (UC-EVs versus AS-EVs). Notably, 86 lipid species were enriched in UC-EVs, with PI 40:6, LPC 18:3/0:0, LPC 20:5/0:0 having the highest FC (6.09, 5.43 and 4.86 respectively). Two-thirds of the deregulated species were enriched in AS-EVs, with PC O-41:3, PE O-41:5 and DG 37:4 having the highest fold change (15.22, 13.84 and 13.44, respectively) (S3 Table).

## Discussion

The need of robust and early detectable disease biomarkers, coupled to the growing interest in EVs cargoes and their appealing application in clinical settings, drove a strong development of the research field associated to EVs, with a focus not only on their biological and clinical role, but also on the technical challenges posed, starting from EVs efficient isolation, characterization and classification. The isolation of EVs from human plasma with a high yield is indeed technically challenging, due to their dimension, the availability of small amounts of starting material and the co-isolation of typical plasma-related vesicular contaminants (e.g. lipoproteins). Moreover, the choice of the isolation method has been reported to be of great importance and must take account of the biological material used, as well as of the technical and functional drawbacks [35, 66–68].

Literature reported that LDL and HDL may be copurified with EVs collected by ultracentrifugation from both serum and plasma [48, 69, 70]. Indeed, HDL, LDL, intermediate-density lipoproteins (IDL), very low-density lipoproteins (VLDL), chylomicrons and other components, like protein aggregates, protein-phospholipid micelles, cell and membrane fragments or intact cells overlap with EVs in terms of size or density and could be co-isolated in EVs fraction [39, 48, 71]. Moreover, multiple pre-analytical variables can affect the quality and the yield of the obtained EVs, such as the time delay between blood collection and processing, the manipulation during transportation and the storage [35, 38–42]. Although the anticoagulant is a main variable to account to, that can influence the downstream application of the samples, little is known about the effect on EVs of anticoagulants used during blood collection and the evidence in literature reported discordant results [38, 39, 41, 43].

In the present paper, a comparison study between UC-EVs and AS-EVs from plasma citrate and plasma EDTA was performed. EVs were isolated by UC, being the most used method with the well-known advantages and disadvantages, and by AS, a well standardized high-throughput method requiring just 30 minutes for obtaining EVs preparations and thus representing a considerable advantage in studies involving a large number of samples. Moreover, AS is easily coupled to RNA and miRNA extraction and analysis, proving to be a useful method for downstream applications [72, 73]. Both UC-EVs and AS-EVs were successfully characterized by NTA, western blot and MACSPlex analysis, and no significant differences were observed between EVs derived from plasma with different anticoagulants, but purified with the same method. Notably, UC- and AS-EVs were detected by TEM, even if their overall quality was not entirely preserved after the freezing/thawing cycle. However, the obtained EVs were adequate for the downstream analysis performed in the present work.

NTA analysis showed an enrichment of smaller particles in UC-EVs pools compared with AS-EVs, suggesting the isolation, by the two methods, not only of different EVs population, but likely of contaminants, as well, as suggested by the EVs/protein ratio lower than 3*10^10^. In particular, lipoproteins, which are critical plasma components, should be taken into consideration. The detection by western blot analysis of APO A1 supported the enrichment in HDL-like particles in UC-EVs. AS-EVs, instead, showed an enrichment in bigger particles. NTA detected a peak around 200 nm, compatible with exosomes and microvesicles, and an increased number of particles around 300–400 nm, that could be represented by chylomicrons. Western blot analysis showed an APO B48/B100 band in AS-EVs, consistent with LDL, VLDL and chylomicrons contamination. Although the presence of larger particles in AS-EVs has been previously reported and attributed to the elution buffer [66], our results are consistent with other studies showing the presence of exosome- and microvesicle-like particles in AS-EVs together with a sub-optimal purification of the EVs due to plasma protein and lipoproteins [66, 68, 72].

The semi-quantitative evaluation of 37 different EVs surface epitopes by using the MACSPlex FACS analysis confirmed that EVs populations isolated from plasma are not affected by the anticoagulant, but, instead are strongly impacted by the purification method. Indeed, UC-EVs appeared as a heterogeneous population, showing both a high variety of cell-specific surface markers (e.g. leukocytes, platelets, endothelium, etc.) and EV-associated tetraspanins. On the other hand, AS-EVs showed a remarkable enrichment of markers specific for the exosomal- microvesicular-compartment, in particular tetraspanins CD9, CD63, and CD81. Moreover, the surface markers profile suggests the preferential isolation of EVs released by leukocytes and, to a lesser degree, by platelets. These findings are coherent with previous reports suggesting the preferential isolation of a certain subclass of EVs by AS [66, 68] and deepen the understanding of their features. Notably, in our experimental conditions, AS-EVs pools showed a certain degree of inhomogeneity in surface epitopes expression. The low reproducibility of MACSPlex analysis in these samples could be a consequence of the high salt concentration of the AS elution buffer, which required a further step of column-based desalting for the downstream MACSPlex analysis. Although desalting made possible MACSPlex analysis, sample quality remained low, reducing reproducibility of FACS analysis.

EVs lipids are emerging as a new important source of biomarkers of disease and health [23, 27]. To evaluate the impact of both anticoagulant and purification methods on EVs lipid composition, we performed a lipidomic analysis by using an untargeted approach. More than 350 lipid species were identified, a number consistent with that reported in previous studies [74, 75]. Analysis of normalized areas revealed that EVs pools derived from plasma with different anticoagulants but purified by the same method had an almost identical lipidomic profile, with few single lipid species enriched in EVs_cit or EVs_EDTA. In particular, LPC16:0/0:0 and LPC 18:0/0:0 enriched in AS-EVs_cit, and LPE 16:00/0:0 enriched in UC-EVs_EDTA were previously reported in the Vesiclepedia database and in literature [75–78]. In the study of Serna and colleagues [77] LPC 18:0 has been found to be particularly enriched in LDL, supporting the hypothesis of a prominent contamination from these lipoproteins in AS-EVs.

Comparison of AS- and UC-EVs lipidomic profiles without considering the type of anticoagulant showed a predominance of GL and FA in UC-EVs, coherent with the contamination from plasma lipoprotein, in particular from HDL, as suggested also by NTA and western blot [79, 80].

Lipid species enriched in AS-EVs were represented by SM, LPE, and PC. Among these, SM are well known structural components of biological membranes involved in the biogenesis of the EVs and one of the most abundant classes reported in EVs [70, 75, 81–83]. SM and PC have also been reported to be enriched in cell culture-derived EVs [84], where they could play both a structural role, increasing the EVs rigidity, and a functional one, acting on their recognition and internalization. Altogether, these observations support the actual isolation of EVs-like particles by AS, albeit technical pitfalls could affect their downstream analysis and applications.

In the present work, we reported the preliminary analysis of a small cohort of subjects, examined as homogeneous pools of healthy males. Surprisingly, the five analyzed pools showed a wide degree of variability in number and dimensions of EVs that could be ascribed to the plasma features of each single subject. However, the main purpose of this report was to define an experimental workflow for the isolation of an unbiased population of EVs useful and scalable to large cohort studies and multiple downstream applications. In the future, the application of this experimental setting to a large cohort of subjects could be implemented for the identification of interpersonal biological variables that were not investigated in here.

In conclusion, in this paper we demonstrate that AS is slightly more efficient than UC in isolating EVs from plasma, but likely results in the selection of a certain sub-population of EVs, restricting the analyzed population. Moreover, we reported technical pitfalls with the downstream analysis of the AS-EVs due to the composition of the elution buffer of the kit most likely. Our data supported previous observations and concerns about the choice of the EVs isolation methods for clinical applications and biomarker discovery [66, 68, 72, 85, 86].

This study contributed to the characterization of the EVs isolated from plasma and supported the notion that a critical evaluation of the EVs isolation method is still necessary.

## Supporting information

S1 File (DOCX)

S1 Fig Size distribution of the EVs isolated by UC from citrate and EDTA pooled plasma. Size distribution (nm) of the isolated EVs using NTA. The insert shows a representative TEM image of PoolA EDTA UC-EVs. Scale bar 100 nm.
(TIF)

S2 Fig Size distribution of the EVs isolated by AS from citrate and EDTA pooled plasma. Size distribution (nm) of the isolated EVs using NTA. The insert shows a representative TEM image of PoolA EDTA AS-EVs. Scale bar 100 nm.
(TIF)

S3 Fig Analysis of EVs surface epitopes detected by MACSPlex kit in UC- and AS-EVs. (A) Western blot showing the EVs marker CD63 and histones in UC- and AS-EVs. A nuclear extract of HCT116 cells was used as positive control for Histones. Box plot showing the normalized MFI of the 28 markers shared by UC-EVs_cit and UC-EVs_EDTA (10–90 percentile) (B) and of the 19 markers shared by AS-EVs_cit and AS-EVs_EDTA (10–90 percentile) (C). Markers on the right side of the dotted line are plotted in the right y-axis.
(TIF)

S4 Fig Analysis of EVs lipid species. Venn diagrams showing the overlap of the lipid species identified by untargeted lipidomic analysis in UC-EV_cit (A), UC-EV_EDTA (B), AS-EV_cit (C) and AS-EV-EDTA (D).
(TIF)

S5 Fig Lipid profile comparison between UC- and AS-EVs. (A) Percentage of lipid species isolated from plasma citrate and plasma EDTA in UC- (blue and dotted-blue respectively) and in AS-EVs (red and dotted-red respectively). (B) Distribution of lipid categories (relative intensities) present in UC- (blue and dotted-blue) and AS-EVs (red and dotted-red) isolated from plasma citrate and plasma EDTA.
(TIF)

S1 Table Markers analyzed by MACSPlex. (XLSX)

S2 Table Lipids classes and species identified by untargeted lipidomic analysis. (XLSX)

S3 Table Significantly deregulated lipid species identified with Metaboanalyst. (XLSX)
