# Supplementary material for: Extracellular vesicles from human plasma for biomarkers discovery: Impact of anticoagulants and isolation techniques
Source: PLoS One. 2023 May 10;18(5):e0285440. doi: 10.1371/journal.pone.0285440 (PMC10171685; doi:10.1371/journal.pone.0285440)
Supplement: S1 File — (DOCX) [file pone.0285440.s001.docx]

**Material and methods supporting information S1**

**Reagents**

For the analysis, LC-MS-grade solvents and reagents were used. Formic acid, ammonium formate and tert-butyl methyl ether were acquired from Merck (Darmstad, Germany), water and acetonitrile were from VWR (Milano, Italy), and methanol was from Scharlab (Barcelona, Spain). As internal standards for lipidomics analysis we employed the SPLASH Lipidomix® [PC 15:0-18:1(d7); PE 15:0-18:1(d7); PS 15:0-18:1(d7); PG 15:0-18:1(d7); PG 15:0-18:1(d7); PA 15:0-18:1(d7); LysoPC 18:1(d7); LysoPE 18:1(d7); Chol Ester 18:1 (d7); 18:1(d7) MG; DG 15:0-18:1(d7); TG 15:0-18:1(d7)-15:0; SM 18:1(d9); Cholesterol (d7)]; DG 12:0-12:0 (Avanti Polar Lipids, Alabaster, AL, USA); and 12-[[(cyclohexylamino)carbonyl]amino]-dodecanoic acid (CUDA) (Cayman Chemicals, Ann Arbor, MI, USA). The following antibodies were used for western-blot analysis of EVs lysates: CD9 (HI9a, BioLegend), CD63 (H5C6, BioLegend), CD81 (5A6, BD Biosciences), Histone (H11-4, Merck), HSP70 (5A5, Santa Cruz Biotechnology), ApoA1 (Wt20-7, Merck), ApoB (2G11, Merck), albumin (HSA-11, Merk).

**Participants, ethical approval and blood sample processing**

To reduce the impact of food intake on circulating EVs, blood was collected in the morning from fasted subjects and consisted in 5 mL drawn in a sodium-citrate plastic tube (BD Vacutainer) and 5 mL drown in an EDTA plastic tube (BD Vacutainer). Blood was drawn into blood collection tubes using a 21 Gauge needle, starting from EDTA tubes. Each Vacutainer was gently inverted several times to mix blood and anticoagulants, as the manufacturer prescribed.

All blood samples were processed by UPO Biobank specialized operators. Blood samples were centrifuged at 500 g for 15 min at room temperature with brake on. From each tube, plasma was transferred into a 15 mL sterile tube, taking care to not disturb the buffy coat layer by stopping the aspiration 1 mL above the buffy coat. The platelet poor plasma (PPP) was obtained by centrifugation at 1500 g for 10 minutes at room temperature with brake on, aliquoted into sterile cryovials and stored at -80°C for at least 4 weeks.

**MACSPlex Human Exosome Kit**

EVs isolated by both UC and AS were subjected to a bead-based flow-cytometry analysis using the MACSPlex Exosome Kit human (Miltenyi Biotec) following the manufacturer’s instruction. Briefly, isolated EVs (20 µg/sample diluted to 120 µL with MACSPlex Buffer) were incubated for 18 hours with the MACSPlex Exosome Capture Beads on an orbital shaker (450 rpm) at room temperature protected from light. Samples were washed with MACSPlex buffer and incubated with the MACSPlex exosome detection reagent mix (APC-conjugated anti-CD9, anti-CD63, and anti-CD81) for 1 hour on an orbital shaker (450 rpm) at room temperature protected from light. Finally, beads were washed twice with the MACSPlex buffer.

For data analysis, using the FlowJo software package (Tree Star Inc., Ashland, OR, USA) and Graphpad Prism 7.0 version, both the background and the mIgG reaction control signals were subtracted from the median fluorescence intensity (MFI) of each marker-specific population. For each anticoagulant and separation method, markers were considered significantly expressed when their MFI was positive in at least 3 out of 5 EVs-pools. Positive MFI values were then normalized by using the mean MFI of all the expressed markers and showed as fold increase.

**Lipids extraction from EVs and from plasma**

For lipids extraction with the biphasic method [61], 200 µL of EVs suspensions (or 30 µL of plasma) were lysed by ultrasound assisted method for 12 seconds. Then, 225 µL of cold methanol containing a mix of deuterated standards (Splash Lipidomix®) were added. The solution was vortexed for 10 sec, followed by the addition of 750 µL of cold MTBE (methyl tert-butyl ether) and vortexed for 10 sec. The tube was then placed in a thermomixer at 4°C and shacked for 6 min at 2000 rpm, followed by the addition of 100 µL of water. The tube was vortexed for 10 sec and then centrifuged for 2 minutes at 14000 rpm at 4°C. Finally, 500 µL of supernatant were collected and evaporated using a SpeedVac concentrator.  The dried sample was reconstituted with 50 µL of a 9/1 (v/v) methanol/toluene solution containing the internal standard CUDA (12.5 ng/mL).

**Untargeted lipidomic analysis**

For the separation of lipids, mobile phase A for the ESI positive mode consisted of acetonitrile/water 60/40 (v/v) with ammonium formate (10 mM) and 0.1% formic acid, while mobile phase B (mpB) contained isopropanol/acetonitrile 90/10 (v/v) with ammonium formate (10 mM) and 0.1% formic acid. In the negative ESI mode, the organic solvents for both mobile phases were the same as for positive ESI mode, with the exception of using ammonium acetate (10 mM) as mobile-phase modifier. The gradient used was as follows: 0–2 min from 30% to 43% mpB, 2–2.1 min from 43% to 55% mfB, 2.1–12 min from 55% to 65% mpB, 12–18 min from 65% to 85% mpB, 18–20 min from 85% to 100% mpB; 100% mpB was kept for 5 min and then the column was allowed to re-equilibrate at 30% mpB for another 5 min. The total run time was 30 min.

The source voltage for mass spectrometry was maintained at 3.5 kV in the positive ion mode and 2.8 kV in the negative ion mode. All other interface settings were identical for the two types of analysis. The capillary temperature was set at 320°C, whereas sheath gas flow and auxiliary gas flow were set at 40 and 3 instrumental arbitrary units, respectively. S-lens was settled at 50 rf. Data were collected in a data-dependent (ddMS2) top 10 scan mode. Survey full-scan MS spectra (mass range m/z 80 to 1200) were acquired with resolution R = 70000 and AGC target 1×10^6^. MS/MS fragmentation was performed using high-energy c-trap dissociation (HCD) with resolution R = 17500 and AGC target 1×10^5^. The stepped normalized collision energy (NCE) was set to 15, 30, and 45, in order. The injection volume was 3 µL.

**Lipidomic data processing**

The cut off value of 85% is based on 6 different similarity scores: one for retention time, one for m/z, one for isotopic pattern, and three for MS/MS (dot product, dot product reversed and presence). Peaks corresponding to internal standards were removed from MS-Dial detected features and were analyzed in the Skyline program to evaluate the reproducibility. The dataset containing m/z values, retention time, peak area, and annotation from the aligned files were exported as an Excel file and manually checked in order to eliminate signals from blanks or wrong records.

Since the determination of cholesterol in biological fluids normally requires a dedicated method, often with a derivatization process before analysis [69], only fatty acids (FA), glycerolipids (GL), glycerophospholipids (GPL) and sphingolipids (SL) were considered.

The Student’s *t*-test was used to evaluate significant differences between citrate and EDTA UC-EVs and between citrate and EDTA AS-EVs. Fisher test was used to compare the observed number of species for each class with that expected by chance. A lipid species was considered significantly modulated when characterized by FC >1.3 and p-value <0.05.
