# Supplementary figures and images for: Extracellular vesicles from human plasma for biomarkers discovery: Impact of anticoagulants and isolation techniques
Source: PLoS One. 2023 May 10;18(5):e0285440. doi: 10.1371/journal.pone.0285440 (PMC10171685; doi:10.1371/journal.pone.0285440)

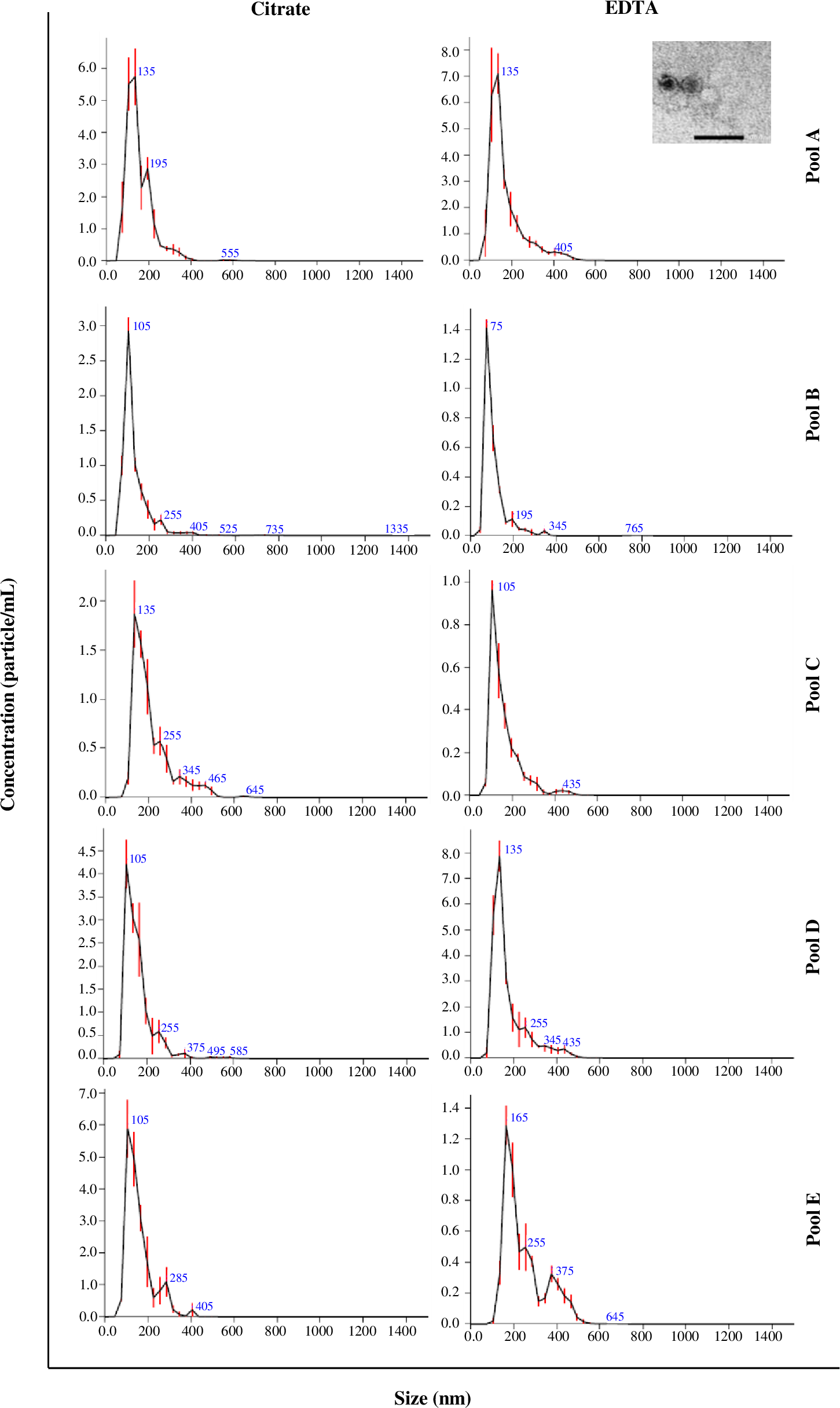

Supplement: S1 Fig — Size distribution (nm) of the isolated EVs using NTA. The insert shows a representative TEM image of PoolA EDTA UC-EVs. Scale bar 100 nm. (TIF) [file pone.0285440.s002.tif]

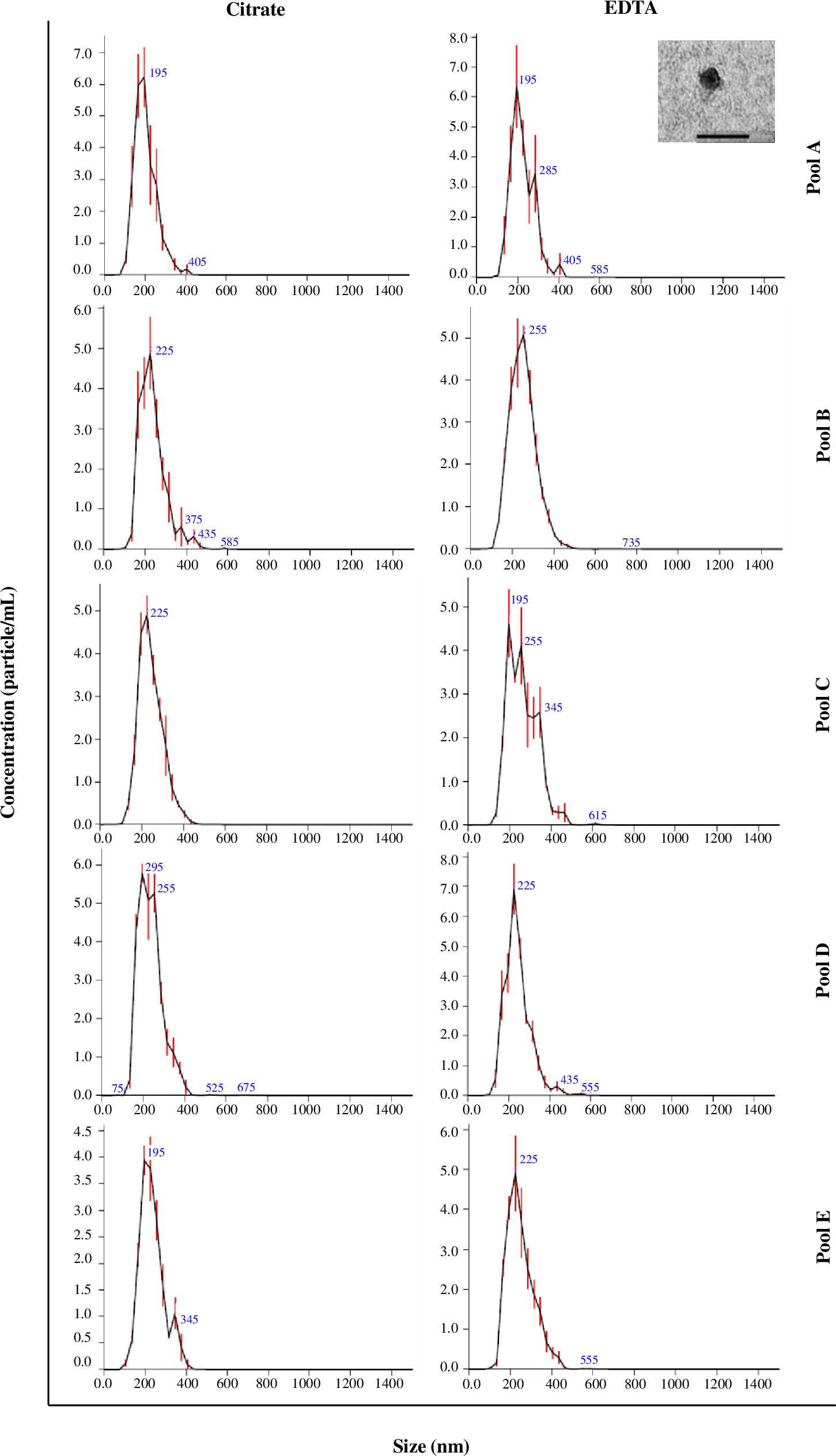

Supplement: S2 Fig — Size distribution (nm) of the isolated EVs using NTA. The insert shows a representative TEM image of PoolA EDTA AS-EVs. Scale bar 100 nm. (TIF) [file pone.0285440.s003.tif]

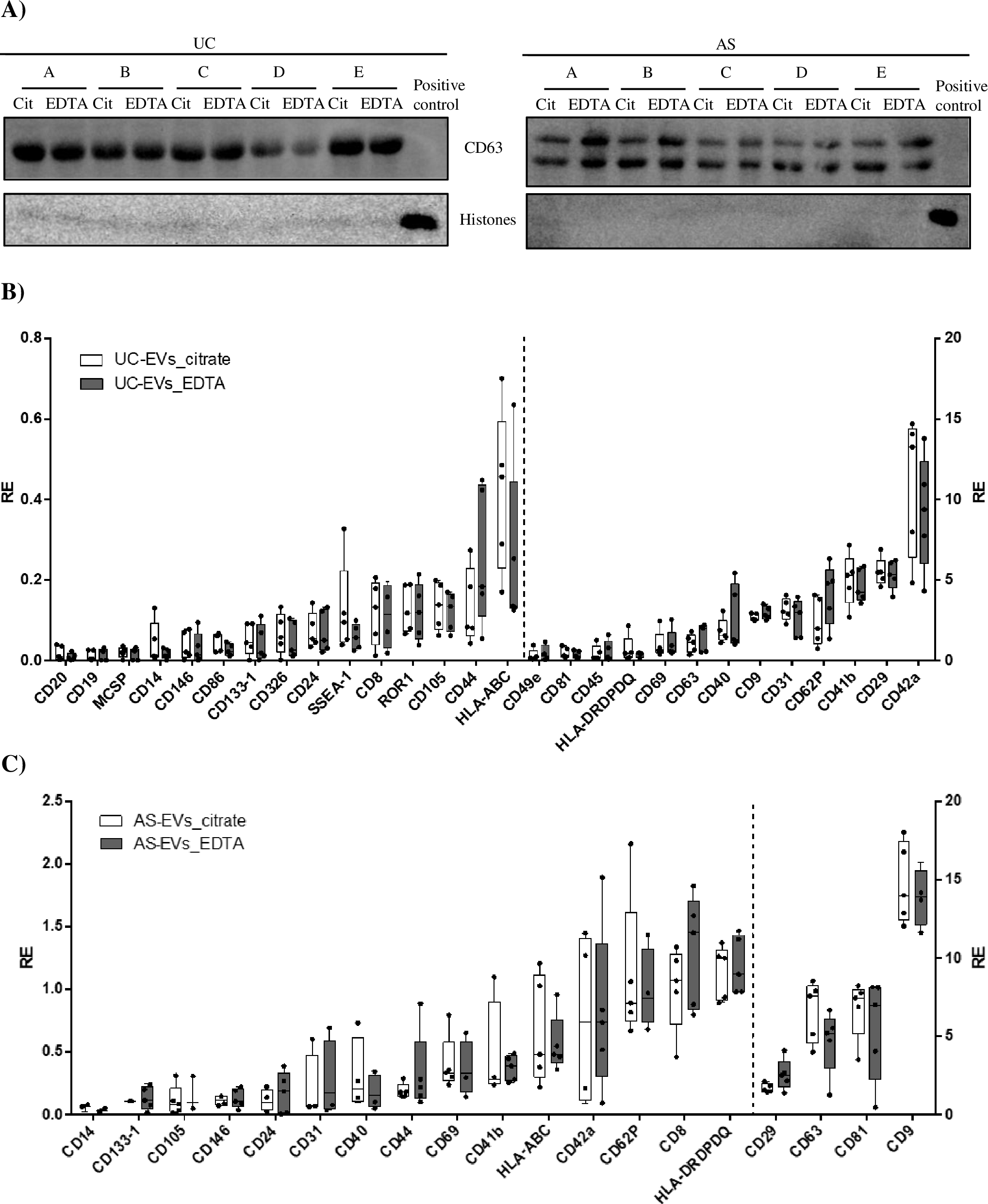

Supplement: S3 Fig — (A) Western blot showing the EVs marker CD63 and histones in UC- and AS-EVs. A nuclear extract of HCT116 cells was used as positive control for Histones. Box plot showing the normalized MFI of the 28 markers shared by UC-EVs_cit and UC-EVs_EDTA (10–90 percentile) (B) and of the 19 markers shared by AS-EVs_cit and AS-EVs_EDTA (10–90 percentile) (C). Markers on the right side of the dotted line are plotted in the right y-axis. (TIF) [file pone.0285440.s004.tif]

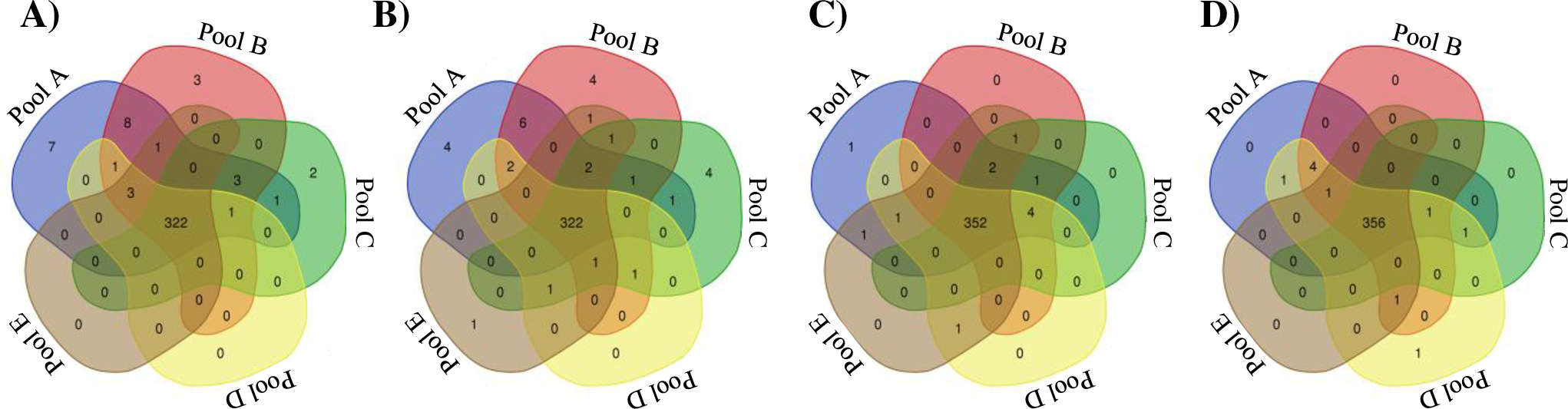

Supplement: S4 Fig — Venn diagrams showing the overlap of the lipid species identified by untargeted lipidomic analysis in UC-EV_cit (A), UC-EV_EDTA (B), AS-EV_cit (C) and AS-EV-EDTA (D). (TIF) [file pone.0285440.s005.tif]

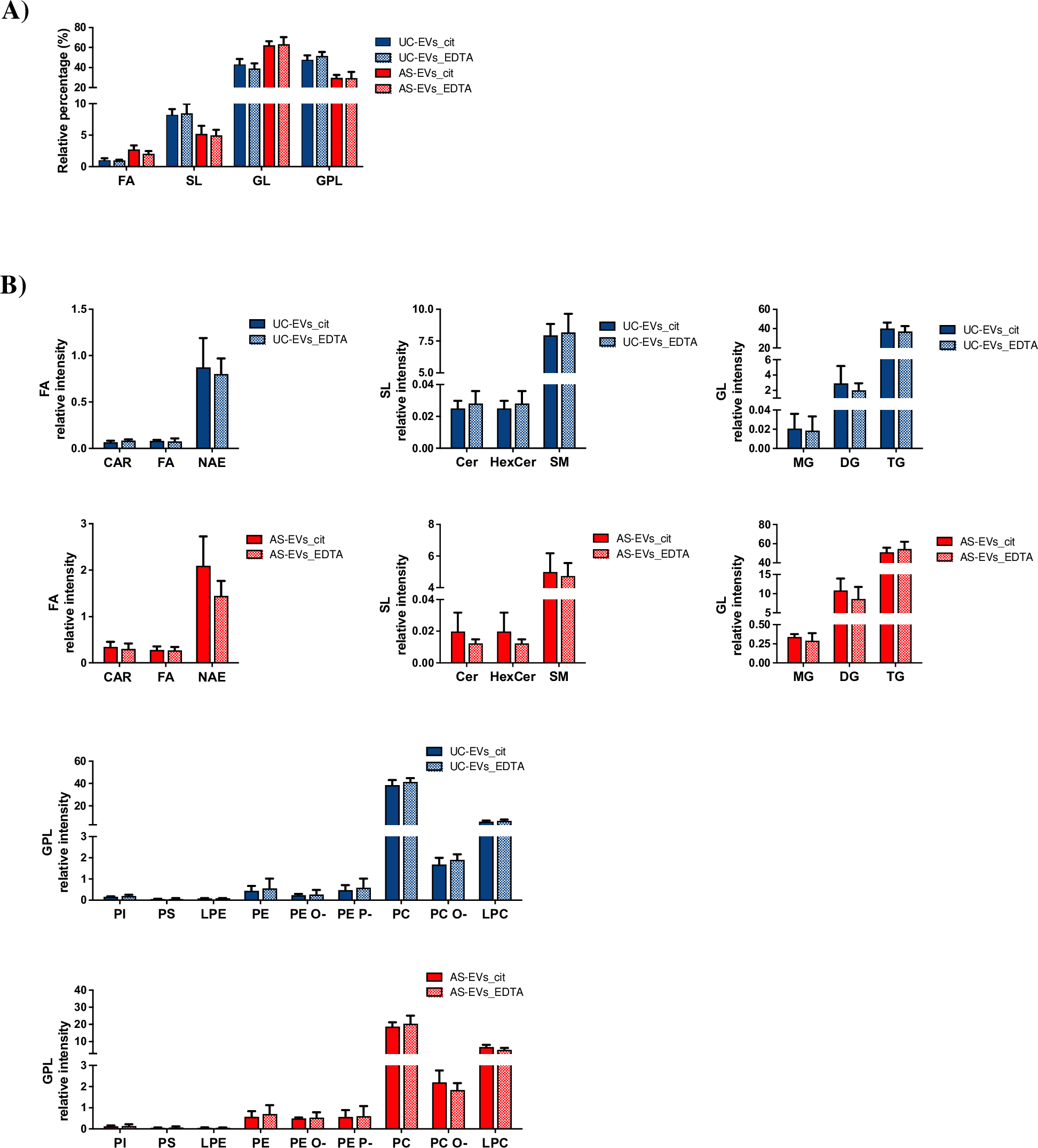

Supplement: S5 Fig — (A) Percentage of lipid species isolated from plasma citrate and plasma EDTA in UC- (blue and dotted-blue respectively) and in AS-EVs (red and dotted-red respectively). (B) Distribution of lipid categories (relative intensities) present in UC- (blue and dotted-blue) and AS-EVs (red and dotted-red) isolated from plasma citrate and plasma EDTA. (TIF) [file pone.0285440.s006.tif]
